# Social disconnection and subsequent mental disorders: a population-based cohort study

**DOI:** 10.1007/s00127-026-03046-y

**Published:** 2026-02-06

**Authors:** Katrine Brandt Alsner, Lisbeth Mølgaard Laustsen, Mathias Lasgaard, Marie Stjerne Grønkjær, Oleguer Plana-Ripoll

**Affiliations:** 1 Department of Clinical Epidemiology, Aarhus University and Aarhus University Hospital, Aarhus, Denmark; 2 DEFACTUM – Public Health Research, Central Denmark Region, Aarhus, Denmark; 3 CORE – Copenhagen Research Center for Mental Health, Copenhagen University Hospital – Bispebjerg and Frederiksberg, Copenhagen, Denmark; 4 Department of Psychology, University of Southern Denmark, Esbjerg, Denmark; 5 Center for Clinical Research and Prevention, Copenhagen University Hospital – Bispebjerg and Frederiksberg, Copenhagen, Denmark; 6 National Centre for Register-based Research, Department of Public Health, Aarhus University, Aarhus, Denmark; 7 ISGlobal, Barcelona, Spain

**Keywords:** Mental health, Mental disorders, Social network, Epidemiology, Risk factors, Public health

## Abstract

**Purpose:**

Social disconnection has been linked to adverse health outcomes, including higher risks of mental disorders. However, previous studies have primarily focused on depression, with limited exploration of other mental disorders and demographic variations. This study investigates the association between social disconnection and a range of subsequent mental disorders in a large, population-based cohort.

**Methods:**

A cohort study was conducted using data from 162,483 participants of the Danish National Health Survey, linked to national health registers. Social disconnection was assessed through survey measures of loneliness, social isolation, and low social support. Incident cases of mental disorders were identified using hospital-based diagnoses and included in seven categories. Poisson regression was applied to estimate incidence rate ratios (IRRs) adjusted for demographics, country of birth, and socio-economic resources.

**Results:**

Individuals who were socially disconnected had a higher incidence rate of mental disorders in all seven categories: substance use disorders, schizophrenia spectrum disorders, bipolar disorder, major depressive disorder, neurotic and anxiety-related disorders, personality disorders, and a combined category of any aforementioned disorder. Loneliness overall showed the strongest associations (range of IRRs, 2.94 to 4.94) compared to social isolation (range of IRRs, 1.47 to 4.80) and low social support (range of IRRs, 1.32 to 2.82). While associations were generally similar across sexes, contrasting age trends were indicated for loneliness and social isolation.

**Conclusion:**

Strong associations were consistently found between social disconnection and subsequent mental disorders, highlighting the potential for targeted public health interventions. Future research should investigate causal mechanisms and directional relations to refine prevention strategies.

**Supplementary Information:**

The online version contains supplementary material available at 10.1007/s00127-026-03046-y.

## Introduction

The field of social disconnection has received growing interest, with research demonstrating that social disconnection such as loneliness, social isolation, and low social support is linked with a range of adverse health outcomes and higher all-cause mortality [1–3]. Individuals who are socially disconnected have a higher risk of a broad range of subsequent medical conditions [4, 5], including coronary heart disease and stroke [6], dementia [7], and diabetes mellitus [8]. Studies have also found that individuals who are socially disconnected have a higher risk of mental disorders such as depression [9–11], depressive symptoms [12], psychosis [13], anxiety-related disorders [14, 15], substance use disorders [16, 17], and personality disorders [18], as well as symptoms of poor mental health such as higher levels of perceived stress [19] and self-harm [20]. While most studies have provided cross-sectional evidence for associations between social disconnection and mental health outcomes [21], there is evidence pointing towards causal links [2]. Possible mechanisms include *psychological* (e.g., stress, resilience, affectivity, and identity formation), *biological* (e.g., neuroendocrine/physiological factors, inflammation, and immune function), and *behavioral* pathways (e.g., physical activity, diet, and sleep) [2]. A recent study applying Mendelian randomisation found evidence for a bidirectional link between loneliness and depression [22]. Besides bidirectional or even cyclical effects, confounding by shared aetiology—such as heritability and adverse life events—may underlie aspects of both social disconnection and mental health outcomes [2, 23].

Most prior studies have focused on depression or depressive symptoms [2, 21] or solely on loneliness rather than examining multiple aspects of social disconnection [9], thereby impeding comparisons across different mental disorders and different aspects of social disconnection. Furthermore, studies have often been constrained by methodological issues including representativeness of the study population and sample size [23]. A large sample size is particularly important for evidence concerning mental disorders with low incidence rates such as schizophrenia and bipolar disorder. Since the prevalence of social disconnection varies across the lifespan [24], exploration of results according to demographic characteristics is also warranted. Enhancing our understanding of the risk of mental disorders among individuals who are socially disconnected is critical for informing both clinical and public health interventions. Thus, there is a need to investigate the association between different indicators of social disconnection and a range of mental disorders in a large representative sample.

The aim of this study was to investigate to what degree three distinct aspects of social disconnection (loneliness, social isolation, and low social support), and a composite measure of these factors, are associated with a range of subsequent mental disorders, and to explore differences with regard to sex and age.

## Methods

### Study population

We carried out a cohort study on a subsample of participants from the Danish National Health Survey, linking with national register data, as described in previous studies [4, 25]. The Danish National Health Survey is conducted every fourth year in five regionally stratified random samples and one national random sample of citizens aged 16 years and above residing in Denmark [26]. Our potential study population consisted of 162,604 individuals who participated in the survey in 2013 (Central Denmark Region) or 2017 (Central Denmark Region, North Denmark Region, Region Zealand, and Capital Region of Denmark), for which data on social disconnection was collected. The response rate for these regions was 57.5%. To account for selection probabilities and non-participation in the survey, we applied inverse probability weights constructed by Statistics Denmark. These weights are made available with the survey data for use in statistical analyses and are based on information from national registers such as sex, age, municipality of residence, educational level, ethnic background, hospitalisations, and occupational status [26]. Due to the population-based sample, 2.1% of the survey responses were provided by individuals who participated in both the 2013 and 2017 survey. To retain the sample for which the population weights were estimated, both responses for these individuals who participated twice were included. Unique identification numbers from the Danish Civil Registration System [27] were employed to link national register data with the survey data. We were able to link a total of 162,483 individuals in the registers at the time of survey participation. A flowchart outlining the definition of the study population is shown in Figure S1.

### Social connections

Loneliness, social isolation, and low social support were assessed using survey data from the Danish National Health Survey. Loneliness can be defined as an unpleasant emotional experience resulting from a perceived lack of social contact [28]. Loneliness was assessed using the Danish version of the Three-Item Loneliness Scale [29, 30], which yields a score ranging from 3 to 9, with higher scores indicating greater loneliness. A score of 7 or above was classified as indicative of loneliness, aligned with the highest threshold identified in previous studies [31]. One of the items was slightly rephrased in 2017 compared to 2013 to improve alignment with the definition of loneliness (the phrasing in Danish of the item ‘How often do you feel left out?’ was slightly changed, excluding one word, to better align with the definition of loneliness by not implying intentional exclusion). When compared to responses in 2013, we found higher values in 2017 for the mean score on this item (1.28 vs. 1.15), the correlation between this item and the scale based on the other two items (item-rest correlation of 0.65 vs. 0.55), and the internal consistency of the scale (Cronbach’s α of 0.81 vs. 0.75). Social isolation pertains to the objective characteristics of an individual’s social connections, indicating a limited network or lack of social contacts [32]. Drawing inspiration from the Berkman-Syme Social Network Index [33] and subsequent adaptions by Valtorta and colleagues [34], social isolation was assessed by quantifying various aspects of social contact. Specifically, four indicators of limited social contact were used, resulting in a score ranging from 0 to 4, based on whether an individual: (i) was living alone, (ii) was unemployed and not enrolled in education, (iii) had less than monthly contact with friends, and (iv) had less than monthly contact with family outside of the household. A score of 3 or above was classified as indicative of social isolation as pre-defined in the analysis plan. Social support covers several forms of perceived and received support. In this study, we assessed perceived emotional support, defined as the perceived availability of verbal care, acceptance, and emotional reciprocity [35]. Drawing inspiration from the MOS Social Support Instrument [36], low social support was assessed using the single-item: “Do you have someone to talk to if you have problems or need for support?” with four response options: “Yes, always”; “Yes, mostly”; “Yes, sometimes”; and “No, never or almost never”. Responses in the last two categories were classified as indicative of low social support as pre-defined in the analysis plan. Finally, we developed a binary composite measure encompassing whether the above measures indicated either loneliness, social isolation, or low social support, thereby capturing both structural and functional aspects of social disconnection. The Danish questionnaires are available at Open Science Framework (https://osf.io/38yru/).

### Mental disorders

Mental disorders were assessed using seven categories with diagnoses based on the International Classification of Diseases, 10th revision (ICD-10): substance use disorders (F10–F19), schizophrenia spectrum disorders (F20–29), bipolar disorder (F30–31), major depressive disorder (F32–F33), neurotic and anxiety-related disorders (F40–F48), personality disorders (F60), and a category including any of the before-mentioned mental disorders. In Denmark, individuals can access publicly funded mental health care either through their general practitioner (GP), who can also prescribe medication, or via emergency visits to psychiatric hospital departments. For non-emergency psychiatric hospital admissions (inpatient or outpatient) and consultations with private practice psychiatrists, a referral from a GP is typically required. In this study, we applied hospital-based diagnoses from inpatient admissions, outpatient visits, and emergency contacts to psychiatric hospital departments (i.e., secondary healthcare utilisation) recorded in the Danish hospital registers [37, 38]. Since emergency contact and outpatient psychiatric visit data have been recorded in these registers since 1995, we included diagnoses in 18 years preceding survey participation to ensure complete data coverage. Accordingly, pre-existing cases (i.e., with onset before survey participation) were defined based on hospital-based diagnoses of mental disorders within the specific category in 18 years preceding survey participation and excluded from the analyses for not being at risk of an incident case. During follow-up (i.e., from the date of survey participation), the onset of a mental disorder was defined as the first date of hospital contact with a diagnosis within the specific category. As an alternative operationalisation of pre-existing mental disorders, we also included information on self-reported mental disorders from the Danish National Health Survey and information on any mental disorder (ICD-10: F00–F90), consultation by a private practice psychiatrist, and psychopharmacological prescriptions in the 18 years preceding survey participation (see Methods S1).

### Covariates

Age, sex (registered legal sex classified as male or female), country of birth (Denmark and Greenland vs. abroad), and linkage to legal parents were obtained from the Danish Civil Registration System [27]. The highest educational level was obtained from the Population Education Register. Income and wealth were retrieved from the Income Statistics Register using the annual disposable equivalised household income and the equivalised household wealth in the calendar year preceding survey participation, adjusted for inflation. For individuals aged 16–29 years, we used their parents’ highest educational level and an average of parental values for income and wealth. Pre-existing physical diseases were assessed using hospital diagnoses from the Danish National Patient Registry and redeemed prescriptions for disease-specific medications from the Danish National Prescription Register [39]. Pre-existing physical diseases were quantified with a disability burden score based on 31 different physical diseases weighted by the disability weights developed by the Global Burden of Disease Study, as described before [40, 41]. Details are provided in Methods S1 and Table S1.

### Statistical analysis

A cohort design was applied for each category of mental disorders, including individuals without any of the specific mental disorders within that category in 18 years preceding survey participation. For each category of mental disorders, individuals were followed from the date of survey participation (2013 or 2017) until a mental disorder diagnosis within that category, death, emigration, or end of data availability (December 31, 2022), whichever came first.

Multiple imputation by chained equations were used to avoid potential bias by excluding 23,036 (14.2%) individuals with partially missing register and/or survey data (see Methods S2). For baseline characteristics of the cohort, we computed means, standard deviations (SDs), and proportions. For all estimates, except absolute numbers, we applied inverse probability of participation weights and used Rubin’s Rules to pool multiple imputed data.

For each aspect of social disconnection, Poisson regression models with Taylor-linearized variance estimation and 95% confidence intervals (CIs) were used to compare the incidence rates of mental disorders between individuals who were socially disconnected (i.e., reporting loneliness, social isolation or low social support) at baseline with those who were not. Two adjustment models were applied. Model 1 estimated the incidence rate ratio (IRR) after adjustments for demographics (age, sex, and year of survey participation). In Model 2, we additionally adjusted for country of birth and socio-economic resources (educational level, income, and wealth). Details regarding adjustment procedures are provided in Methods S1. In stratified analyses, we investigated whether these associations varied according to sex and baseline age. Formal statistical tests of these subgroup differences were not conducted; accordingly, the observed patterns are based on differences in point estimates rather than tests of pre-specified hypotheses.

Three sensitivity analyses were performed. First, to assess potential confounding by pre-existing health conditions, we adjusted for pre-existing physical diseases using a summarised disability burden score [40]. Second, to assess the unclear temporality between social disconnection and mental disorders, we investigated if similar results were obtained if we excluded individuals based on an alternative operationalisation of pre-existing mental disorders, while at the same time delaying the start of follow-up to six months after the survey participation. This alternative operationalisation of pre-existing mental disorders included self-reported information, any hospital diagnosis, redeemed prescriptions, and consultations with private practice psychiatrists who received subsidies from the public health insurance (see Methods S1). Finally, to explore the employed thresholds for loneliness, social isolation, and low social support, we examined the results for specific scores/responses.

All statistical analyses were conducted in Stata version 18.0 using the svy and mi suite of commands. A preregistered analysis plan is available at Open Science Framework (https://osf.io/38yru/). Due to programming constraints, we were unable to account for intra-individual correlation among the 2.1% of respondents who participated twice. However, our previous study [25] based on the same study population showed that cluster-robust standard errors yielded nearly identical results.

## Results

The mean age at survey participation of the 162,483 participants was 48.3 years (SD 19.1), and 87,619 (50.6%) were women. The number of individuals who were classified as ‘lonely’ was 9,817 (7.6%), while 4,713 (3.5%) were classified as ‘socially isolated’, and 21,380 (14.9%) as having ‘low social support’. Among individuals with at least one of the indicators of social disconnection, 26% had more than one indicator (Figure S2). Baseline characteristics of the study population stratified by the classifications of social disconnection are presented in Table 1. At baseline, the proportion of individuals with hospital-diagnosed and self-reported mental disorders, psychopharmacological redemptions, and consultations with a private practice psychiatrist was 2- to 4-fold higher among those classified with loneliness, social isolation and low social support compared to those without these indicators. Furthermore, unhealthy lifestyle behaviours regarding sleep, smoking, alcohol consumption, and physical inactivity were more prevalent among individuals who were socially disconnected. The number of pre-existing cases at baseline ranged from 658 for bipolar disorder to 9,047 for any of the included mental disorders, and the number of new cases during follow-up ranged from 231 for bipolar disorder to 2,818 for any of the included mental disorders (Table S2). Over the follow-up period, 11,165 individuals died, and 2,696 individuals emigrated.

**Table 1:** Baseline characteristics of the cohort in four regions of Denmark, 2013 and 2017

	Lonely: *N* = 9,817 (7.6%)	Socially isolated: *N* = 4,713 (3.5%)	Low social support: *N* = 21,380 (14.9%)	Neither lonely, socially isolated, or low social support: *N* = 134,197 (80.0%)
Age, mean (SD)	43.3 (25.6)	64.4 (27.9)	48.1 (24.7)	48.2 (23.0)
Women, N (%)	5,826 (55.1)	2,180 (44.4)	10,440 (45.0)	73,175 (51.3)
Survey participation in 2013 as opposed to 2017, N (%)	1,245 (13.6)	900 (19.3)	4,241 (20.1)	27,959 (22.2)
Born abroad, N (%)	1,558 (23.0)	470 (14.6)	2,979 (21.4)	8,423 (10.2)
Educational level				
Lowest (ISCED 0–2), N (%)	3,054 (33.8)	2,121 (49.9)	6,084 (31.2)	28,981 (23.8)
Middle (ISCED 3–5), N (%)	4,640 (45.5)	1,902 (37.3)	10,580 (47.3)	65,746 (48.0)
Highest (ISCED 6–8), N (%)	2,122 (20.7)	690 (12.7)	4,716 (21.5)	39,470 (28.2)
Annual disposable equivalised household income (1,000 DKK), mean (SD)	238.5 (231.9)	192.8 (127.5)	249.9 (213.7)	296.2 (312.9)
Equivalised household wealth (1,000 DKK), mean (SD)	316.2 (2,119.6)	516.3 (2,381.9)	451.4 (2,067.1)	573.2 (3,759.7)
Disability burden score, mean (SD)	0.2 (0.2)	0.3 (0.3)	0.2 (0.2)	0.2 (0.2)
The Three-Item Loneliness Scale, mean (SD)	7.8 (1.2)	5.4 (2.8)	5.4 (2.6)	3.6 (1.2)
The adapted Social Isolation Index				
Living alone, N (%)	3,699 (40.3)	3,544 (79.1)	6,245 (33.5)	21,309 (19.1)
Out of employment and not enrolled in education N (%)	4,585 (44.8)	4,475 (93.7)	8,878 (40.0)	46,482 (30.9)
Less than monthly contact with friends, N (%)	2,506 (24.9)	3,474 (73.3)	4,304 (20.4)	7,212 (5.2)
Less than monthly contact with family N (%)	1,896 (20.0)	3,167 (66.5)	3,680 (18.7)	6,121 (5.0)
The social support item				
Social support never or almost never available, N (%)	1,945 (20.5)	962 (22.2)	6,641 (32.3)	0 (0)
Social support sometimes available, N (%)	3,249 (32.5)	1,062 (23.2)	14,739 (67.7)	0 (0)
Social support mostly available, N (%)	2,798 (28.4)	1,240 (25.6)	0 (0)	39,878 (30.2)
Social support always available, N (%)	1,824 (18.6)	1,449 (29.0)	0 (0)	94,319 (69.8)
Pre-existing hospital-diagnosed mental disorder, N (%)	2,381 (25.9)	855 (21.2)	2,610 (14.0)	6,675 (5.9)
Self-reported pre-existing or current mental disorder, N (%)	4,367 (45.5)	1,339 (32.1)	5,510 (27.4)	16,543 (13.2)
Psychopharmacological redemption, N (%)	5,034 (48.7)	2,412 (52.1)	8,050 (36.5)	32,640 (23.0)
Consultation with a private practice psychiatrist, N (%)	1,653 (17.0)	582 (13.5)	2,097 (10.3)	5,670 (4.4)
Not getting enough sleep to feel rested, N (%)	3,226 (33.2)	897 (20.7)	4,777 (23.7)	11,921 (9.6)
Daily smoking*, N (%)	2,082 (23.9)	1,184 (28.0)	4,147 (21.7)	18,479 (15.4)
Weekly units of alcohol*, mean (SD)	8.0 (21.4)	11.4 (27.6)	8.4 (19.2)	7.2 (11.6)
Weekly binge drinking (≥ 5 units on the same occasion)*, N (%)	1,008 (15.0)	482 (16.6)	2,357 (15.7)	12,364 (12.3)
One or less days per week with physical activity (≥ 30 min)**, N (%)	1,008 (32.1)	593 (37.0)	2,079 (26.1)	8,593 (16.7)

Missing data was imputed using multiple imputation by chained equations. Absolute numbers are unweighted, whereas means, standard deviations, and percentages are weighted based on register data to represent the population of the included regions in 2013 and 2017; accordingly, the weighted percentages do not match the absolute numbers. Note that loneliness, social isolation, and low social support are not mutually exclusive; therefore, the percentages in the top row do not sum up to 1.

*Based on complete rather than imputed survey data

**Based on complete rather than imputed survey data from a subsample

**Fig. 1:**
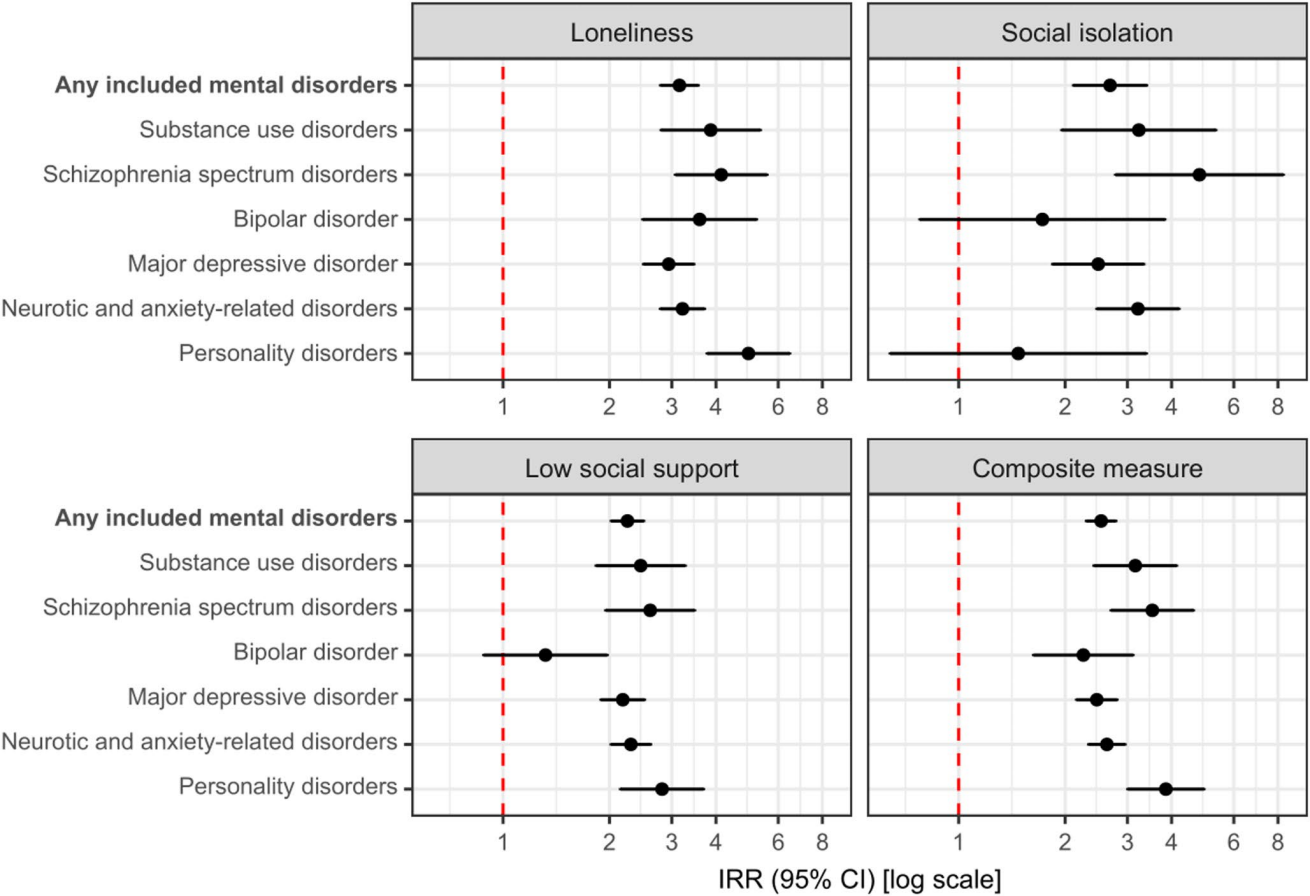
Social disconnection and relative differences in incidence rates of seven mental disorder categories in four regions of Denmark, 2013–2022. CI: Confidence interval; IRR: Incidence rate ratio. Missing data was imputed using multiple imputation by chained equations, and the results are weighted based on register data to represent the population of the included regions in 2013 and 2017. All estimates are adjusted for age, sex, year of survey participation, country of birth, educational level, income, and wealth (Model 2) and are provided in Table S3

Figure 1 provides relative differences in the incidence rates of the seven mental disorder categories according to social disconnection from Model 2, which showed a similar pattern as those from Model 1 (Table S3). Individuals with social disconnection, compared to those without, had higher rates of subsequent mental disorders in all seven categories with the highest IRRs for schizophrenia spectrum disorders, substance use disorders, and personality disorders. Out of the three different aspects of social disconnection, the highest IRRs were overall found for loneliness with a median IRR of 3.60 (range, 2.94 to 4.94), whereas the median IRR was 2.68 (range, 1.47 to 4.80) for social isolation and 2.30 (range, 1.32 to 2.82) for low social support. The strongest pairwise associations, with IRRs above 4, were found for loneliness or social isolation and subsequent schizophrenia spectrum disorders and for loneliness and subsequent personality disorders. The weakest pairwise associations, with IRRs below 2, were found for social isolation or low social support and a subsequent bipolar disorder and for social isolation and subsequent personality disorders; however, these estimates had a high degree of uncertainty.

**Fig. 2:**
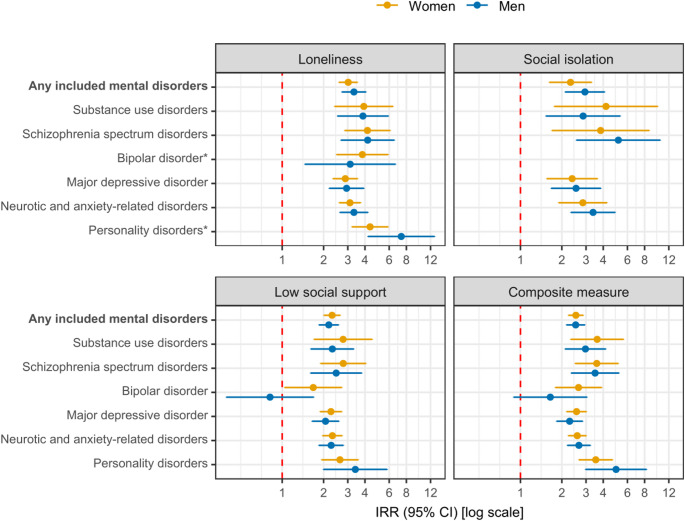
Social disconnection and sex-stratified relative differences in incidence rates of seven mental disorder categories in four regions of Denmark, 2013–2022. CI: Confidence interval; IRR: Incidence rate ratio. Missing data was imputed using multiple imputation by chained equations, and the results are weighted based on register data to represent the population of the included regions in 2013 and 2017. All estimates are adjusted for age, year of survey participation, country of birth, educational level, income, and wealth (Model 2) and are provided in Table S4. *Sex-stratified results for social isolation and a subsequent bipolar or personality disorder are not provided due to few cases

Figure 2 provides sex-stratified relative differences in the incidence rates of the seven mental disorder categories according to social disconnection from Model 2. When comparing point estimates, the IRRs were similar for women and men, with a median IRR of mental disorders for the composite measure of 2.65 (range, 2.53 to 3.61) for women and 2.66 (range, 1.65 to 4.96) for men. A sex difference was indicated for low social support and a subsequent bipolar disorder although with some uncertainty (IRR of 1.68 [95% CI, 1.04 to 2.72] for women vs. 0.82 [95% CI, 0.39 to 1.70] for men). A particularly high IRR was found for loneliness and subsequent personality disorders for men (7.35 [95% CI, 4.20-12.84]).

**Fig. 3:**
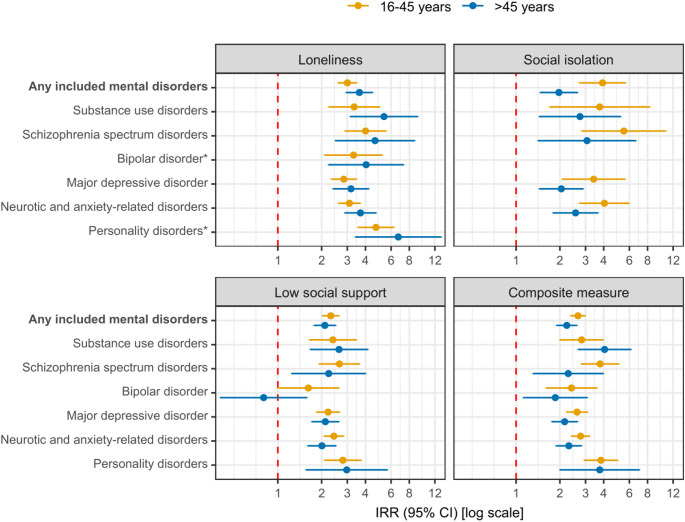
Social disconnection and age-stratified relative differences in incidence rates of seven mental disorder categories in four regions of Denmark, 2013–2022. CI: Confidence interval; IRR: Incidence rate ratio. Missing data was imputed using multiple imputation by chained equations, and the results are weighted based on register data to represent the population of the included regions in 2013 and 2017. All estimates are adjusted for age, sex, year of survey participation, country of birth, educational level, income, and wealth (Model 2) and are provided in Table S5. *Age-stratified results for social isolation and a subsequent bipolar or personality disorder are not provided due to few cases

Figure 3 provides age-stratified relative differences in the incidence rates of the seven mental disorder categories according to social disconnection from Model 2. When comparing point estimates for loneliness, individuals aged above 45 years had a higher median IRR of 4.04 (range, 3.18 to 6.72) compared to individuals aged 16–45 years with a median IRR of 3.31 (range, 2.84 to 4.72). When comparing point estimates for social isolation, however, individuals aged above 45 years had a lower median IRR of 2.56 (range, 1.97 to 3.07) compared to individuals aged below 45 years, who had a median IRR of 3.93 (range, 3.41 to 5.50). A similar analysis was conducted across three age groups (16–30 years, 31–45 years, and > 45 years); however, due to low number of events, only results pertaining to the composite measure are presented (Figure S3). In general, we found no systematic differences between the three age groups except for a potential age trend for substance use disorders with higher IRRs among older individuals.

Three sensitivity analyses were performed. The first sensitivity analysis with further adjustment for a summarised disability burden score showed similar IRRs to the main analysis (Figure S4). The second analysis with delayed start of follow-up by six months and exclusion of individuals based on an alternative operationalisation of mental disorders provided slightly attenuated associations for the category pertaining to any of the included mental disorders but increased IRRs for bipolar disorder (Figure S4). In the third sensitivity analysis on the employed thresholds for loneliness, social isolation, and low social support, a dose-response-like association was indicated for loneliness scores, while a threshold-like association was indicated for social isolation scores and social support responses (Figure S5).

## Discussion

Using a population-based cohort study with 162,483 participants from the Danish National Health Survey, this study expands current knowledge on social disconnection and subsequent mental disorders. In general, all three examined aspects of social disconnection (loneliness, social isolation, and low social support) were associated with higher incidence rates for all seven categories of mental disorders, with IRRs typically around 3. The strongest associations were overall observed for loneliness, and the weakest, with IRRs below 2, were observed for associations that were estimated with high uncertainty. We found similar IRRs among women and men, but higher IRRs for loneliness among older individuals in contrast to higher IRRs for social isolation among younger individuals. When excluding individuals based on an alternative operationalisation of mental disorders and delaying the start of follow-up, attenuated IRRs were found for the category with any of the included mental disorders, but, interestingly, increased IRRs were found for bipolar disorder.

Most prior studies on social disconnection and subsequent mental disorders have focused on development of depression following experiences of loneliness [9]. In general, we have found similar or higher estimates compared to those previously reported in large longitudinal studies on depression [10, 11]. However, direct comparisons are challenging due to varying definitions of ‘loneliness’ and different dichotomisation as well as continuous inclusion of the Three-Item Loneliness Scale. Differences are also to be expected given that we examined hospital-diagnosed depression of which the severity may be higher than applied cut-offs on depression scales. Consistent with our results, previous studies have shown that individuals who experience social disconnection have higher rates of anxiety [15], post-traumatic stress disorders [14], psychosis [13], excessive alcohol use [16], opioid use [17], and personality disorders [18]. Few studies have addressed the other mental disorders included in our study. To the best of our knowledge, our study is the first longitudinal study on social disconnection and subsequent schizophrenia spectrum disorders and bipolar disorder given that previous studies [42] have assessed disease progression rather than incident cases.

## Strengths and limitations

Study strengths include the large population-based sample, and the use of almost complete register data extracted from national databases to avoid loss to follow-up. Another strength is the use of rich survey and register data including three distinct aspects of social disconnection, among these a validated measure for loneliness, and seven diagnostic categories for mental disorders with an 18-year look-back period to detect pre-existing cases. Although no systematic validation has been conducted for all mental disorders, the validity has been confirmed for some diagnoses, e.g., schizophrenia, single episode depression, and autism [38].

Our study also carries important limitations, which include potential selection bias in the survey although inverse probability weights and imputation of partially missing data were employed to minimise this risk. There are also important limitations in the measures of social disconnection and mental disorders. The Danish wording of the Three-Item Loneliness Scale may have been less optimal for the subsample who participated in 2013. Although no clear consensus exists on how to quantify social isolation in existing literature [43], the applied social isolation index included employment status but not participation in associational activities or voluntary work and will thus be correlated with socio-economic position. The single-item on social support exclusively captured perceived emotional support rather than other types of support. Although the applied register data on hospital-based diagnoses of mental disorders is considered to have almost full coverage for severe mental disorders such as schizophrenia that are routinely treated in psychiatric hospital departments, individuals who are treated by general practitioners or private practice psychiatrists or individuals who do not seek help, are not included. Studies have estimated that, in Denmark, only 14.5% of individuals who screened positive for depression and 24.7% of individuals who screened positive for anxiety received a hospital-based diagnosis of major depressive disorder [44] or an anxiety-related disorder [45], respectively. Given the possibility of diagnostic delay, undiagnosed mental disorders, and diagnoses registered prior to the 18-year look-back period, the temporal link between social disconnection and mental disorders is uncertain. If such outcome misclassification is associated with social disconnection, this could additionally lead to bias in an unpredictable direction. Given that we only had information on social disconnection at one point in time, we could neither address the duration of these exposures nor determine confounding or mediation by other risk factors. Accordingly, we chose not to adjust for a range of baseline characteristics such as health behaviours and marital status, but we adjusted for socio-economic resources given their established role as key social determinants of health [23].

### Implications

Our findings could suggest that social disconnection precedes mental disorders, although the unclear temporality due to diagnostic delay, undetected diagnoses, and undiagnosed conditions should be taken into account. For example, incident cases of mental disorders that typically have an early onset, such as schizophrenia and personality disorders [46], identified among individuals older than 45 years may reflect earlier onset that was not captured in the registry data. Importantly, given that the study was not designed as a causal study with extensive confounding control, the estimated associations could indicate causal links but could also be explained by residual confounding by, for instance, heritability and adverse life events that may shape personality traits, health behaviour, and cognitive patterns. Notably, age-related differences were observed. For instance, social isolation was more strongly associated with subsequent mental disorders among individuals aged 45 years or younger than among older individuals. This may reflect the inclusion of employment and educational enrolment in the operationalisation of social isolation—factors that carry distinct significance during early adulthood. Accordingly, social isolation may, to a greater extent at younger ages, capture a marginalised group with elevated susceptibility to mental disorders. Although noteworthy, the modest magnitude of the age-related differences suggests limited relevance for clinical decision-making or interventions. Furthermore, the absence of substantial sex differences is consistent with previous research on loneliness, suggesting comparable effects among men and women [9]. This finding indicates that the well-established sex differences in the incidence of mental disorders [46] are likely attributable to factors other than social disconnection.

The findings of this study provide evidence of strong and consistent associations between three distinct aspects of social disconnection and subsequent diagnoses of a range of mental disorders. This knowledge can enable clinicians and public health planners to more accurately identify the preventive needs of individuals who are socially disconnected. Furthermore, it can provide valuable insights into the potential role of social disconnection in the overall burden of mental disorders. While prior research suggests a causal link between social disconnection and an increased risk of depression [2], the directionality and causality of associations with substance use, schizophrenia, bipolar, anxiety, and personality disorders remain uncertain [21]. Further research is needed to clarify the directionality and causality and to explore the underlying psychological, biological, and behavioural mechanisms that mediate or confound these associations [2]. In particular, studies leveraging repeated measures and advanced causal inference techniques could help disentangle these associations. If social disconnection plays a causal role in the onset of mental disorders, this underscores an opportunity for intervention—particularly because these social risk factors are modifiable. Interventions have demonstrated moderate effectiveness in reducing loneliness [47] and social support [48]; however, further research is needed to establish who such interventions would help the most and investigate long-term impact.

## Conclusion

This study provides evidence that social disconnection—characterised by loneliness, social isolation, and low social support—is associated with a wide range of subsequent mental disorders. Individuals who were socially disconnected had a higher incidence rate of substance use disorders, schizophrenia spectrum disorders, bipolar disorder, major depressive disorder, neurotic and anxiety-related disorders, personality disorders, and a combined category of any aforementioned disorder with loneliness overall showing the strongest associations. Differences in risk patterns were indicated across age groups, with older individuals showing stronger associations with loneliness and younger individuals showing stronger associations with social isolation. Further research is needed to elucidate underlying mechanisms and assess the directional nature of these associations.

## Supplementary Information

Below is the link to the electronic supplementary material.

Supplementary Material 1

## Data Availability

Data presented in this study were obtained from Danish registries and regions participating in the Danish National Health Survey. Owing to data protection rules, we are not allowed to share individual-level data. Other researchers who fulfil the requirements set by the data providers may gain access to the data through Statistics Denmark, the Danish Health Data Authority and/or the Danish regions (Central Denmark Region, North Denmark Region, Region Zealand and Capital Region of Denmark). A preregistered analysis plan is available at Open Science Framework (https://osf.io/38yru/).
