# Supplementary material for: Social disconnection and subsequent mental disorders: a population-based cohort study
Source: Soc Psychiatry Psychiatr Epidemiol. 2026 Feb 6;61(5):925–36. doi: 10.1007/s00127-026-03046-y (PMC13156090; doi:10.1007/s00127-026-03046-y)
Supplement: Supplementary file 1 — Supplementary Material 1 [file 127_2026_3046_MOESM1_ESM.pdf]

Supplementary appendix

This appendix has been provided by the authors to give readers additional information about the following paper:

Social disconnection and subsequent mental disorders: a population-based cohort study

Authors: Katrine Brandt Alsner*, Lisbeth Mølgaard Laustsen*, Mathias Lasgaard, Marie Stjerne Grønkjær, Oleguer Plana-Ripoll
*Shared first authors

Contents

Supplementary Methods **2**

Methods S1. Definition of and adjustment for covariates 2

Methods S2. Multiple imputation 4

Supplementary Figures **9**

Figure S1. Flowchart depicting the initial study population in four regions of Denmark, 2013 and 2017 9

Figure S2. Venn diagram of the distribution of loneliness, social isolation, and low social support among the initial study population in four regions of Denmark, 2013 and 2017 9

Figure S3. The composite measure of social disconnection and age-stratified relative differences in incidence rates of seven mental disorder categories in four regions of Denmark, 2013-2022 10

Figure S4. Sensitivity analyses of social disconnection and relative differences in incidence rates of seven mental disorder categories in four regions of Denmark, 2013-2022 11

Figure S5. Specific scores/responses on social disconnection and relative differences in incidence rates of any of the included mental disorders in four regions of Denmark, 2013-2022 12

Supplementary Tables **13**

Table S1. List of diagnosis and prescription codes in each physical disease category and associated disability weights 13

Table S2. Persons at risk at start of follow-up and cases during follow-up in the total cohort of 162,483 individuals in four regions of Denmark, 2013-2022 16

Table S3. Social disconnection and relative differences in incidence rates of seven mental disorder categories in four regions of Denmark, 2013-2022 17

Table S4. Social disconnection and sex-stratified relative differences in incidence rates of seven mental disorder categories in four regions of Denmark, 2013-2022 18

Table S5. Social disconnection and age-stratified relative differences in incidence rates of seven mental disorder categories in four regions of Denmark, 2013-2022 19

References **20**

Supplementary Methods

Methods S1. Definition of and adjustment for covariates

Age

Age was included as a time-varying covariate in 1-year incremental age groups and modelled as a natural cubic spline with 5 knots. For analyses in subgroups based on age group, we remodelled age using a natural cubic spline with 4 knots fitted for the specific age group.

Educational level

To classify educational level, we utilized the International Standard Classification of Education (ISCED) (1). We considered a bachelor's degree or higher (ISCED levels 6–8) as the highest educational level; an upper secondary school, vocational education, or short-cycle tertiary education (ISCED levels 3–5) as a middle educational level; and up to secondary school (ISCED levels 0–2) as the lowest educational level.

Income and wealth

To allow for comparisons across different household sizes, income and wealth were both retrieved for the household and equivalised according to the household size using OECD's modified equivalence scale (2). To adjust for inflation, we standardized to values in 2016 with the new gross domestic product deflator from the World Bank (worldbank.org). Income included all registered disposable work income, capital income, and transfers of public benefits after deduction of tax (3). To allow for comparison between homeowners and tenants, the estimated rental value of housing was also included for homeowners, after exclusion of their interest expenses (3). Wealth included all assets and debts in properties, financial institutions, investment deposits, and credit unions, but not pension assets, debt owed to private individuals, investments outside deposits, cash holdings, and other assets such as cars or yachts (4). Income and wealth were modelled as categorical variables using quartiles based on the distribution of values in the study population.

Disability burden score

The disability burden is based on 31 physical diseases belonging to nine overall categories: cardiovascular, endocrine, respiratory, gastrointestinal, urogenital, musculoskeletal, haematological and neurological diseases, and cancers. A previous Danish study demonstrated that, apart from cancers, all nine categories of physical diseases are associated with an elevated risk of mental disorders and indicated a dose-response relation between disability burden score and risk of subsequent mental disorders (5).The disability burden score is a continuous value from 0 (equivalent to no disease burden) and increasing for individuals with multiple simultaneous diseases, with a

potential maximum value at 1. Disability burden score was modelled as a natural cubic spline with 3 knots.

Alternative operationalisations of pre-existing mental disorders

We considered the following as indicators of a pre-existing mental disorders:

i) Self-reported preceding or current mental disorder at baseline in the Danish National Health Survey
ii) Any hospital-diagnosed mental disorder (International Classification of Diseases, 10th revision: F00-F90) in 18 years preceding survey participation
iii) Redeemed prescription for psychopharmacological treatment recorded in the Danish National Prescription Registry in 18 years preceding survey participation, including antipsychotics (N05A except N05AN), antidepressants (N06A), lithium (N05AN), anxiolytics (N05B except N05BA01), medication for ADHD (C02AC02, N06BA02, N06BA04, N06BA09, and N06BA12), and medication for alcohol and opioid dependence (N07BB-N07BB04) based on a recent study (6)
iv) Consultation with a private practice psychiatrist subsidied from the public health insurance and thereby recorded in the Danish National Health Service Register in 18 years preceding survey participation

Methods S2. Multiple imputation

Most individuals with missing data were missing information on one or more survey variables (12.6%) while fewer individuals were missing register data, primarily on educational level (1.9%) as shown in Methods Table 1. To assess whether exclusion of individuals with partly missing data could bias the results, we conducted a quantitative bias analysis (Methods Table 2). We found that missingness on both survey and register data could be associated with the outcome, in both directions, after adjustment for covariates which entail a risk of bias (7,8). Individuals with missing survey data were on average older, whereas individuals with missing register data were on average younger and more likely to have been born abroad (Methods Table 3).

To ensure that the imputation of missing data would add information to the analyses, we included a range of auxiliary variables. We used multiple imputation by chained equations (MICE) with separate imputation models for survey and register data. Each model conducted 15 imputations after a burn-in of 10 and applied predictive mean matching (PMM) using a recommended donor pool of 10 observations (9). Methods Table 4 provides the applied variables for imputation of scores/responses on loneliness, social isolation, social support, and self-reported mental disorder. Methods Table 5 provides the applied variables for imputation of educational level, income, wealth, and country of birth. We included the analysis model variables such as the outcome, censoring variables, and population weights in the imputation models, as recommend (10,11). To account for the use of parental values as a proxy for individuals aged below 30 years, we stratified the imputation model for register data by age below 30 years. Methods Table 6 provides the distribution of complete and imputed data.

Methods Table 1: Overview of missing data in the cohort in four regions of Denmark, 2013 and 2017

	**All individuals: N = 162,483**
**Overall**	
Missing on any variable, N (%)	23,036 (14.2)
Missing on survey data, N (%)	20,528 (12.6)
Missing on register data, N (%)	3,081 (1.9)
**Survey data**	
Missing on loneliness, N (%)	9,483 (5.8)
Missing on social isolation, N (%)	13,076 (8.0)
Missing on social support, N (%)	7,151 (4.4)
Missing on self-reported mental disorder, N (%)	11,683 (7.2)
Missing on all survey variables above, N (%)	4,281 (2.6)
**Register data**	
Missing on educational level, N (%)	3,081 (1.9)
Missing on annual disposable equivalised household income, N (%)	1,313 (0.8)
Missing on equivalised household wealth, N (%)	1,313 (0.8)
Missing on all register variables above, N (%)	1,313 (0.8)

Absolute numbers and proportions are unweighted.

Methods Table 2: Quantitative bias analysis among the 162,483 individuals with register linkage, for all categories of mental disorders, in four regions of Denmark, 2013-2022

	**Individuals with complete survey data (N = 141,955)**		**Individuals with complete register data (N = 159,402)**	
	Events/person-years at risk with missing register data	IRR for missing register data (95% CI)*	Events/person-years at risk with missing survey data	IRR for missing survey data (95% CI)**
Substance use disorders	10/13,628	1.46 (0.68–3.12)	55/125,426	1.29 (0.92–1.82)
Schizophrenia spectrum disorders	5/13,666	0.43 (0.14–1.33)	54/125,829	1.22 (0.85–1.75)
Bipolar disorder***	–	–	30/126,249	1.20 (0.77–1.88)
Major depressive disorder	29/13,425	0.67 (0.42–1.07)	208/122,864	1.02 (0.85–1.22)
Anxiety or neurotic-related disorders	28/13,261	0.55 (0.35–0.86)	257/121,483	1.14 (0.97–1.34)
Personality disorders	6/13,634	0.37 (0.15–0.92)	42/125,609	0.97 (0.67–1.42)
Any of the included disorders	50/12,882	0.70 (0.50–0.99)	397/117,796	1.16 (1.02–1.32)

*Adjusted for age, sex, year of survey participation, and the composite measure
**Adjusted for age, sex, year of survey participation, country of birth, educational level, income, and wealth
***Results for bipolar disorder according to missing register data are not provided due to few cases

Methods Table 3: Additional characteristics of the cohort in four regions of Denmark, 2013 and 2017

	**Included in CCA (N = 139,447)**	**Missing survey data (N = 20,528)**	**Missing register data (N = 3,081)**
Age, mean (SD)	47.5 (18.4)	54.7 (21.8)	40.8 (21.9)
Women, N (%)	74,898 (50.2)	11,417 (52.6)	1,612 (51.8)
Survey participation in 2013 as opposed to 2017, N (%)	27,068 (21.0)	5,724 (26.2)	626 (17.6)
Born abroad, N (%)	8,906 (9.7)	1,848 (14.9)	1,768 (70.9)
Living with a partner (self-reported), N (%)	98,001 (64.0)	8,033 (53.7)	1,721 (54.7)
Emotional limitations in daily activities (item 6-7 in SF-12), mean (SD)	8.6 (2.0)	7.9 (2.4)	8.0 (2.3)
The sum of the Perceived Stress Scale, mean (SD)	12.1 (7.3)	13.8 (7.3)	14.4 (7.2)
In education c.f. register data, N (%)	11,177 (11.2)	1,316 (8.6)	361 (18.1)
In employment c.f. register data, N (%)	79,476 (56.9)	7,321 (36.9)	1,172 (41.2)
Cohabitation c.f. register data, N (%)	107,127 (71.6)	13,388 (60.0)	1,759 (51.5)

Missing data was imputed using multiple imputation by chained equations, and percentages are weighted based on register data to represent the population of the included regions in 2013 and 2017.

Methods Table 4: Variables applied in imputation of survey data among individuals in four regions of Denmark, 2013 and 2017

	N (%) missing	Range	Mean (SD)
**Imputed variables**			
The Three-Item Loneliness Scale, item 1	8,469 (5.2)	1 to 3	1.3 (0.5)
The Three-Item Loneliness Scale, item 2	8,494 (5.2)	1 to 3	1.4 (0.6)
The Three-Item Loneliness Scale, item 3	8,909 (5.5)	1 to 3	1.3 (0.5)
The social isolation index, living alone	8,384 (5.2)	0 to 1	0.2 (0.4)
The social isolation index, out of employment and not enrolled in education	9,267 (5.7)	0 to 1	0.4 (0.5)
The social isolation index, less than monthly contact with friends	8,381 (5.2)	0 to 1	0.1 (0.3)
The social isolation index, less than monthly contact with family	7,397 (4.6)	0 to 1	0.1 (0.3)
The social support item	7,151 (4.4)	1 to 4	1.6 (0.8)
Prior mental disorder, self-reported	11,683 (7.2)	0 to 1	0.1 (0.4)
**Analysis model variables**			
Population weights*	0 (0)	1 to 226	29.2 (19.6)
Age**	0 (0)	16 to 103	52.2 (18.4)
Female	0 (0)	0 to 1	0.5 (0.5)
Born abroad	≤5 (NA)	0 to 1	0.1 (0.3)
Year of survey	0 (0)	2,013 to 2,017	2,016.2 (1.6)
Disability burden score**	0 (0)	0 to 1	0.2 (0.2)
Substance use disorder during follow-up	0 (0)	0 to 1	0.002 (0.05)
Schizophrenia and related disorder during follow-up	0 (0)	0 to 1	0.002 (0.05)
Bipolar disorder during follow-up	0 (0)	0 to 1	0.001 (0.04)
Major depressive disorder during follow-up	0 (0)	0 to 1	0.01 (0.1)
Anxiety or neurotic disorder during follow-up	0 (0)	0 to 1	0.01 (0.1)
Personality disorders during follow-up	0 (0)	0 to 1	0.002 (0.05)
Death during follow-up	0 (0)	0 to 1	0.1 (0.3)
Emigration during follow-up	0 (0)	0 to 1	0.02 (0.1)
**Auxiliary variables at survey participation**			
Prior hospital-diagnosed mental disorder	0 (0)	0 to 1	0.1 (0.2)
Prior psychopharmacological redemption	0 (0)	0 to 1	0.3 (0.4)
Prior consultation with private practicing psychiatrist	0 (0)	0 to 1	0.1 (0.2)
Enrolled in education c.f. register data	1,409 (0.9)	0 to 1	0.1 (0.3)
In employment c.f. register data	1,409 (0.9)	0 to 1	0.5 (0.5)
Cohabitation c.f. register data	1,409 (0.9)	0 to 1	0.8 (0.4)
Living with a partner, self-reported	9,238 (5.7)	0 to 1	0.7 (0.5)
Long-term disease, self-reported	7,690 (4.7)	0 to 1	0.4 (0.5)
The sum of the Perceived Stress Scale*	10,716 (6.6)	0 to 40	11.8 (7.1)
Score on getting enough sleep to feel rested	6,467 (4.0)	1 to 3	1.5 (0.7)
Spending time unwanted alone*	7,291 (4.5)	1 to 4	3.2 (0.9)
Evaluation of own health (item 1 in SF-12)*	1,082 (0.7)	1 to 5	2.6 (0.9)
Emotional limitations in daily activities (item 6-7 in SF-12)*	5,074 (3.1)	2 to 10	8.6 (2.0)
Mental health and vitality (item 9-11 in SF-12)*	6,133 (3.8)	3 to 15	9.3 (1.6)
Social contact limited due to physical or mental health (item 12 in SF-12)*	3,188 (2.0)	1 to 5	4.5 (0.9)

NA: Not applicable; SF-12: 12-Item Short Form Survey. Absolute numbers, percentages, means, and SDs are unweighted. The range is shown using means of the 5 lowest and highest values.

*Included in the imputation model as a linear term

**Included in the imputation model as a natural cubic spline with 3 knots

Methods Table 5: Variables applied in imputation of register data among individuals in four regions of Denmark, 2013 and 2017

	**Aged 16-29 years (N = 24,943): Imputation of parental educational level, income, and wealth**			**Aged ≥30 years (N = 137,540): Imputation of own educational level, income, and wealth**		
	N (%) missing	*Range*	*Mean (SD)*	*N (%) missing*	*Range*	*Mean (SD)*
**Imputed variables**						
Parental/own educational level	1,373 (5.5)	1 to 3	2.1 (0.7)	1,708 (1.2)	1 to 3	1.9 (0.7)
Parental/own annual disposable equivalised household income (1,000 DKK)	1,299 (5.2)	-11,201 to 11,696	319.7 (340.1)	14 (0.0001)	-9,683 to 14,935	293.9 (233.2)
Parental/own equivalised household wealth (1,000 DKK)	1,299 (5.2)	-68,863 to 134,658	480.0 (3082.4)	14 (0.0001)	-144,231 to 244,313	686.8 (3124.6)
Born abroad	≤5 (NA)	0 to 1	0.1 (0.3)	≤5 (NA)	0 to 1	0.1 (0.3)
**Analysis model variables**						
Population weights*	0 (0)	2 to 222	42.2 (24.7)	0 (0)	1 to 217	26.8 (17.5)
Mean of parental/own age**	1,309 (5.2)	34 to 77	53.4 (5.8)	0 (0)	30 to 103	57.5 (14.5)
Female	NA	NA	NA	0 (0)	0 to 1	0.5 (0.5)
Calendar year*	0 (0)	2,013 to 2,017	2016.2 (1.6)	0 (0)	2,013 to 2,017	2016.2 (1.6)
Disability burden score*	0 (0)	0 to 1	0.1 (0.1)	0 (0)	0 to 1	0.2 (0.2)
Substance use disorder during follow-up	0 (0)	0 to 1	0.01 (0.1)	0 (0)	0 to 1	0.001 (0.04)
Schizophrenia and related disorder during follow-up	0 (0)	0 to 1	0.01 (0.1)	0 (0)	0 to 1	0.001 (0.03)
Bipolar disorder during follow-up	0 (0)	0 to 1	0.003 (0.1)	0 (0)	0 to 1	0.001 (0.03)
Major depressive disorder during follow-up	0 (0)	0 to 1	0.02 (0.1)	0 (0)	0 to 1	0.01 (0.1)
Anxiety or neurotic disorder during follow-up	0 (0)	0 to 1	0.03 (0.2)	0 (0)	0 to 1	0.01 (0.1)
Personality disorders during follow-up	0 (0)	0 to 1	0.01 (0.1)	0 (0)	0 to 1	0.001 (0.03)
Death during follow-up	0 (0)	0 to 1	0.002 (0.04)	0 (0)	0 to 1	0.1 (0.3)
Emigration during follow-up	0 (0)	0 to 1	0.1 (0.2)	0 (0)	0 to 1	0.01 (0.1)
**Auxiliary variables**						
Prior hospital-diagnosed mental disorder	0 (0)	0 to 1	0.1 (0.3)	0 (0)	0 to 1	0.1 (0.2)
Prior psychopharmacological redemption	0 (0)	0 to 1	0.1 (0.3)	0 (0)	0 to 1	0.3 (0.5)
Prior consultation with private practicing psychiatrist	0 (0)	0 to 1	0.04 (0.2)	0 (0)	0 to 1	0.1 (0.2)
Mother's age at childbirth**	1,809 (7.3)	14 to 47	29.6 (4.7)	NA	NA	NA
Number of children in own/parents' household(s)*	1,299 (5.2)	0 to 7	0.2 (0.5)	14 (0.0001)	0 to 8	0.4 (0.8)
Number of adults in own/parents' household(s)*	1,299 (5.2)	1 to 7	2.6 (0.9)	14 (0.0001)	1 to 7	1.9 (0.7)
Parental/own educational level 5 years prior	1,452 (5.8)	1 to 3	2.1 (0.7)	3,337 (2.4)	1 to 3	1.9 (0.7)

	**Aged 16-29 years (N = 24,943): Imputation of parental educational level, income, and wealth**			**Aged ≥30 years (N = 137,540): Imputation of own educational level, income, and wealth**		
	N (%) missing	*Range*	*Mean (SD)*	*N (%) missing*	*Range*	*Mean (SD)*
Parental/own yearly disposable income 5 years prior (1,000 DKK)*	1,377 (5.5)	-8,467 to 5,927	274.8 (238.6)	1,706 (1.2)	-13,509 to 20,609	273.5 (251.4)
Parental/own current wealth 5 years prior (1,000 DKK)*	1,377 (5.5)	-68,505 to 120,113	424.3 (2897.0)	1,706 (1.2)	-47,956 to 153,617	664.4 (2185.5)
Offspring's yearly disposable income the year prior (1,000 DKK)*	≤5 (NA)	-2,061 to 9,925	217.0 (230.2)	NA	NA	NA
Offspring's current wealth the year prior (1,000 DKK)*	≤5 (NA)	-11,378 to 95,531	157.2 (1991.8)	NA	NA	NA

NA: Not applicable. Absolute numbers, percentages, means, and SDs are unweighted. The range is shown using means of the 5 lowest and highest values.

*Included in the imputation model as a linear term

**Included in the imputation model as a natural cubic spline with 3 knots

Methods Table 6: Distribution of variables in complete and imputed data among individuals in four regions of Denmark, 2013 and 2017

	**Complete data**	**Imputed data**
**Survey data**		
Lonely, N (%)	8,935 (7.3)	882 (11.2)
Socially isolated, N (%)	3,963 (3.2)	750 (6.5)
Low social support, N (%)	20,263 (14.7)	1,117 (17.4)
Self-reported pre-existing or current mental disorder, N (%)	22,386 (16.3)	2,032 (18.6)
**Register data preceding survey participation**		
Educational level		
Lowest (ISCED 0-2), N (%)	36,603 (25.6)	910 (29.5)
Middle (ISCED 3-5), N (%)	77,897 (47.6)	1,475 (48.2)
Highest (ISCED 6-8), N (%)	44,902 (26.9)	696 (22.2)
Disposable annual equivalized household income (1,000 DKK), mean (SD)	286 (251)	273 (277)
Equivalized household wealth (1,000 DKK), mean (SD)	552 (3,069)	371 (2,568)

Absolute numbers are unweighted, whereas means, SDs, and proportions are weighted based on register data to represent the population of the included regions in 2013 and 2017.

Supplementary Figures

Figure S1. Flowchart depicting the initial study population in four regions of Denmark, 2013 and 2017

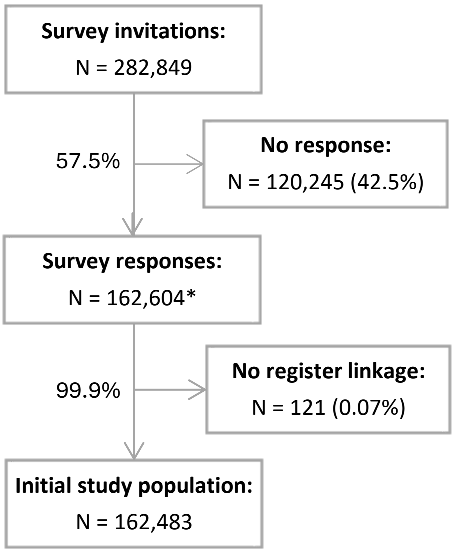

* For a small number (N = 23) of survey participants in 2013, the specific date of survey participation was missing and replaced with the median date.

Figure S2. Venn diagram of the distribution of loneliness, social isolation, and low social support among the initial study population in four regions of Denmark, 2013 and 2017

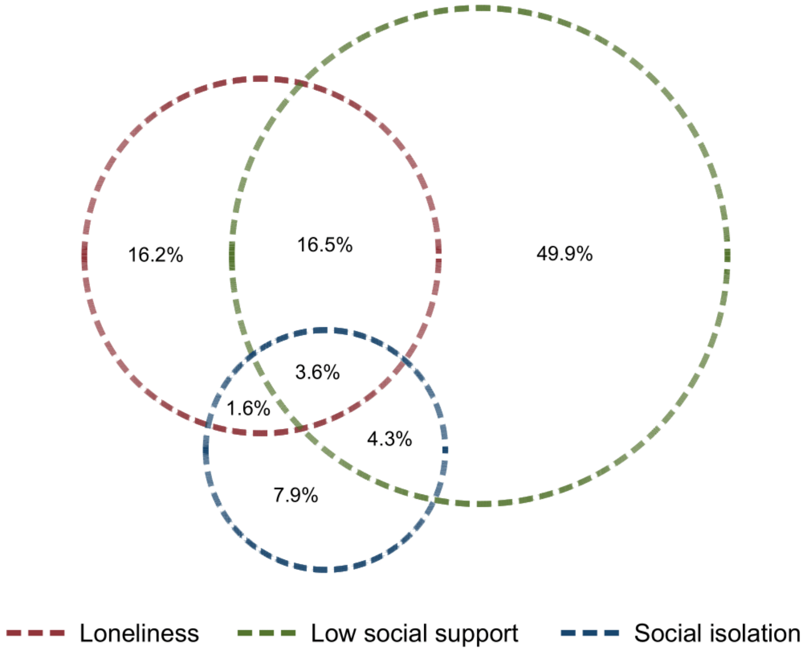

Missing data were imputed using multiple imputation by chained equations. Proportions are shown for individuals with at least one of the indicators of social disconnection and weighted based on register data to represent the population of the included regions in 2013 and 2017.

Figure S3. The composite measure of social disconnection and age-stratified relative differences in incidence rates of seven mental disorder categories in four regions of Denmark, 2013-2022

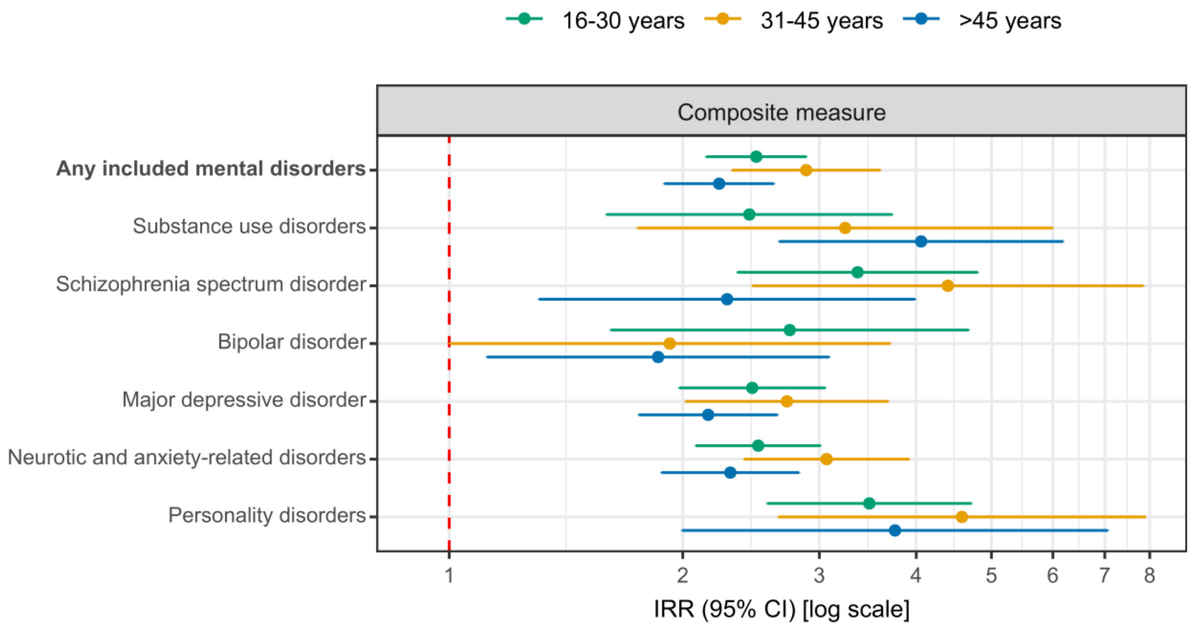

CI: Confidence interval; IRR: Incidence rate ratio. Missing data was imputed using multiple imputation by chained equations, and the results are weighted based on register data to represent the population of the included regions in 2013 and 2017. All estimates are adjusted for age, sex, year of survey participation, country of birth, educational level, income, and wealth (Model 2).

Figure S4. Sensitivity analyses of social disconnection and relative differences in incidence rates of seven mental disorder categories in four regions of Denmark, 2013-2022

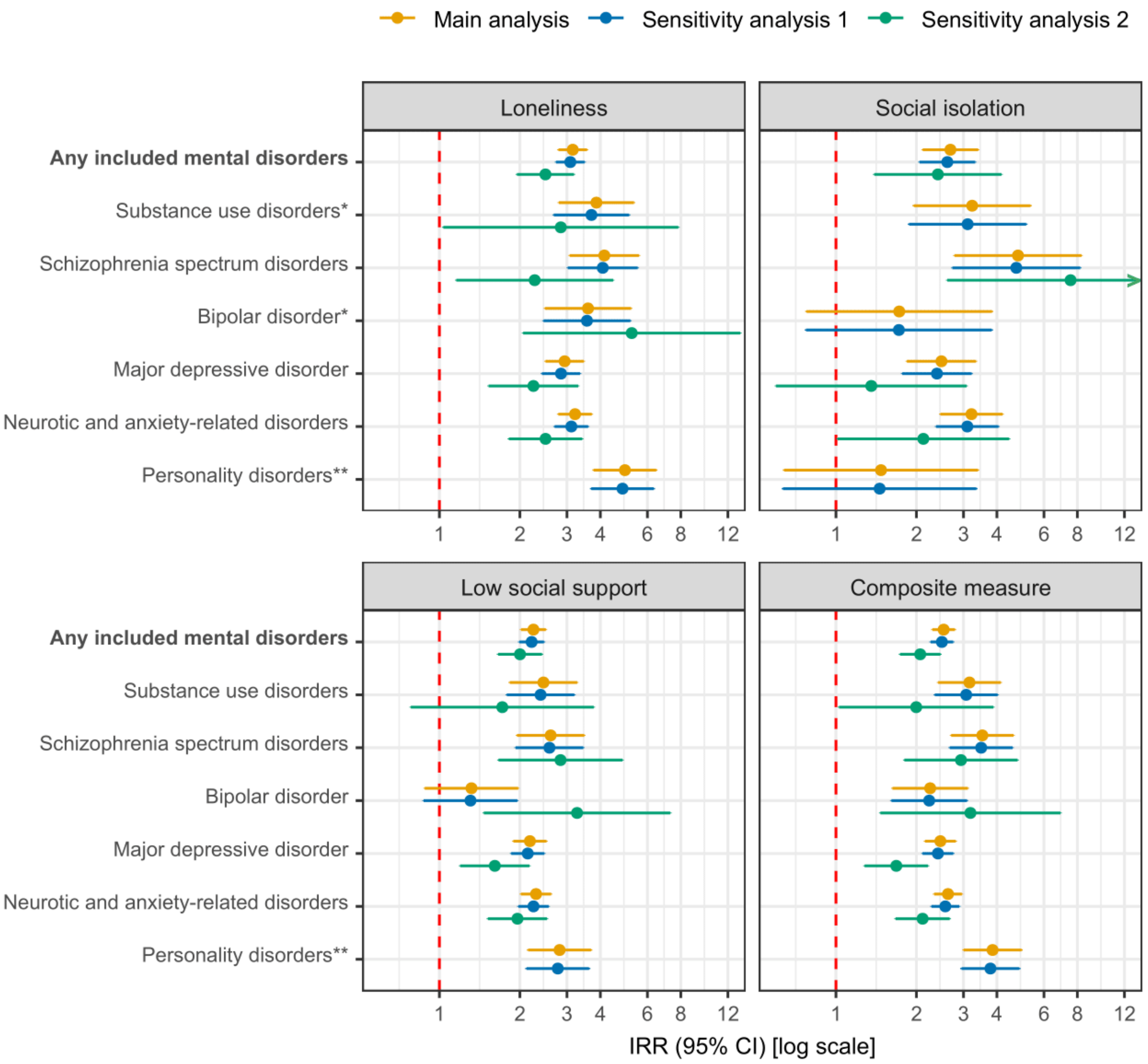

CI: Confidence interval; IRR: Incidence rate ratio. Missing data was imputed using multiple imputation by chained equations, and the results are weighted based on register data to represent the population of the included regions in 2013 and 2017. All estimates are adjusted for age, sex, year of survey participation, country of birth, educational level, income, and wealth (Model 2). The first sensitivity analysis applied further adjustment for a summarised disability burden score, while the second analysis delayed the start of follow-up by six months and excluded individuals based on an alternative operationalisation of mental disorders.

Figure S5. Specific scores/responses on social disconnection and relative differences in incidence rates of any of the included mental disorders in four regions of Denmark, 2013-2022

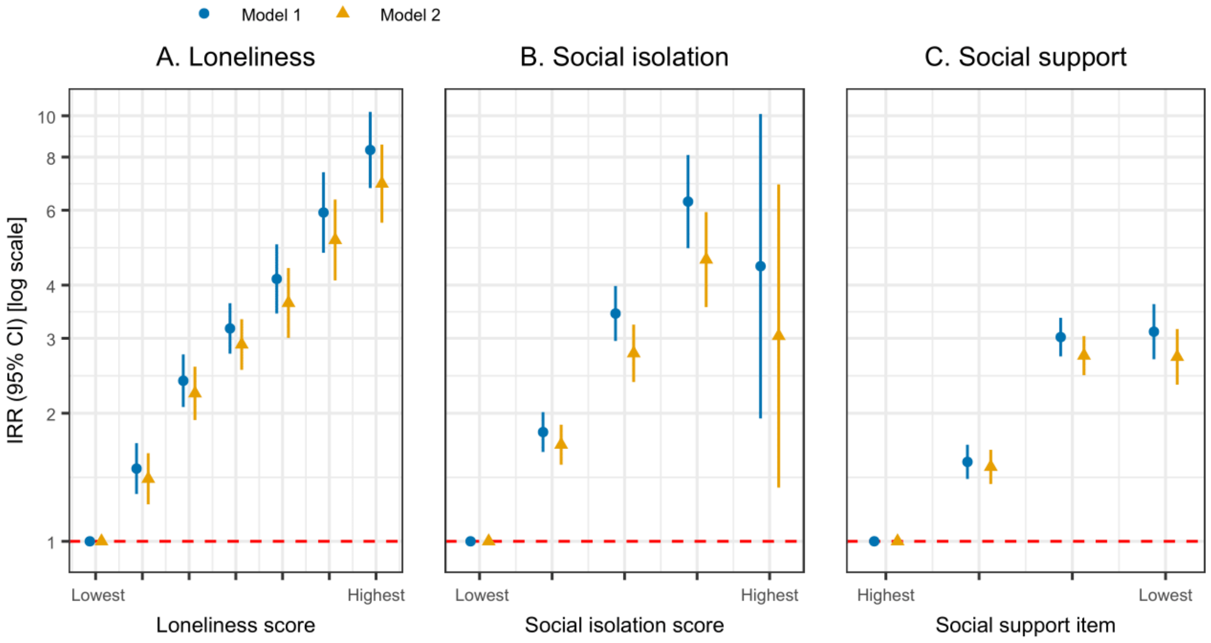

CI: Confidence interval; IRR: Incidence rate ratio. Missing data was imputed using multiple imputation by chained equations, and the results are weighted based on register data to represent the population of the included regions in 2013 and 2017. All estimates are adjusted for age, sex, year of survey participation, country of birth, educational level, income, and wealth (Model 2).

Supplementary Tables

Table S1. List of diagnosis and prescription codes in each physical disease category and associated disability weights

Coding number	Medical condition category	Coding definition	Diagnosis/mortality codes (ICD-10)	Medication		Disability weight (95% confidence interval)
				Drug codes (ATC)	Time frame for prescriptions	
**CIRCULATORY SYSTEM**						
1	Hypertension	Diagnosis AND/OR prescriptions of antihypertensives [a]	I10–I13, I15	C02, C03, C04, C07, C08, C09	Twice in 1 year	0.049 (0.031–0.072)
2	Dyslipidaemia	Diagnosis AND/OR prescription of lipid-lowering drugs [b]	E78	C10	Twice in 1 year	0.049 (0.031–0.072)
3	Ischemic heart disease	Diagnosis AND/OR prescription for antianginal drug	I20–I25	C01DA	Twice in 1 year	0.108 (0.074–0.152)
4	Atrial fibrillation	Diagnosis	I48			0.224 (0.151–0.315)
5	Heart failure	Diagnosis	I50			0.092 (0.062–0.131)
6	Peripheral artery occlusive disease	Diagnosis	I70–I74			0.014 (0.007–0.025)
7	Stroke	Diagnosis	I60–I64, I69			0.104 (0.045–0.181)
**ENDOCRINE DISEASES**						
8	Diabetes mellitus	Diagnosis AND/OR prescription of antidiabetics	E10-E14	A10A, A10B	Twice in 1 year	0.077 (0.051–0.111)
9	Thyroid disorder	Diagnosis AND/OR prescription of thyroid therapy drugs	E00–E05, E061–E069, E07	H03	Twice in 1 year	0.066 (0.040–0.101)
10	Gout	Diagnosis	E79, M10			0.023 (0.014–0.034)
**RESPIRATORY DISEASES**						
1101	Chronic obstructive pulmonary disease	Diagnosis AND/OR Prescription for obstructive airway disease drugs	J40–J44, J47 [c]	R03	Twice in 1 year	0.126 (0.077–0.177)
1102	Asthma	Diagnosis AND/OR Prescription for obstructive airway disease drugs	J45–J46 [c]	R03	Twice in 1 year	0.065 (0.043–0.095)
12	Allergy	Diagnosis AND/OR Prescription for non-sedative antihistamines AND/OR nasal antiallergics	J30.1–J30.4, L23, L50.0, T78.0, T78.2, T78.4 [c]	R06AX, R06AE07, R06AE09, R01AC, R01AD	Twice in 1 year	0.218 (0.138–0.308)

Coding number	Medical condition category	Coding definition	Diagnosis/mortality codes (ICD-10)	Medication		Disability weight (95% confidence interval)
				Drug codes (ATC)	Time frame for prescriptions	
**GASTROINTESTINAL DISEASES**						
13	Ulcer/chronic gastritis	Diagnosis	K221, K25–K28, K293–K295			0.074 (0.040–0.086)
14	Chronic liver disease	Diagnosis	B16–B19, K70–K74, K766, I85			0.178 (0.123–0.243)
15	Inflammatory bowel disease	Diagnosis	K50–K51			0.231 (0.156–0.320)
16	Diverticular disease of intestine	Diagnosis	K57			0.049 (0.031–0.072)
**UROGENITAL DISEASES**						
17	Chronic kidney disease	Diagnosis	N03, N11, N18-N19			0.016 (0.010–0.024)
18	Prostate disorders	Diagnosis AND/OR prescription of prostate hyperplasia therapy drugs	N40	C02CA, G04C	Twice in 1 year	0.067 (0.043–0.097)
**MUSCULOSKELETAL DISEASES**						
19	Connective tissue disorders	Diagnosis	M05–M06, M08–M09, M30–M36, D86			0.131 (0.097–0.160)
20	Osteoporosis	Diagnosis AND/OR prescription for osteoporosis drugs	M80–M82	M05B, G03XC01, H05AA	Twice in 1 year	0.131 (0.097–0.160)
21	Painful condition	Repeated prescriptions of analgesics		N02A, N02BA51, N02BE, M01A, M02A	4x in 1 year	0.159 (0.100–0.222)
**HAEMATOLOGICAL DISEASES**						
22	HIV/AIDS	Diagnosis	B20-B24			0.049 (0.031–0.072)
23	Anaemias	Diagnosis	D50–D53, D55–D59, D60–D61, D63-D64			0.029 (0.018–0.043)
**CANCER**						
24	Cancer	Diagnosis	C00–C43, C45–C97			0.105 (0.102–0.108)
**NEUROLOGIC DISEASES**						
25	Vision problem	Diagnosis	H25, H40, H54			0.085 (0.055–0.121)
26	Hearing problem	Diagnosis	H90–H91, H931			0.029 (0.016–0.045)
27	Migraine	Diagnosis AND/OR prescription of specific anti-migraine drugs	G43	N02C	Twice in 1 year	0.037 (0.017–0.066)

Coding number	Medical condition category	Coding definition	Diagnosis/mortality codes (ICD-10)	Medication		Disability weight (95% confidence interval)
				**Drug codes (ATC)**	**Time frame for prescriptions**	
28	Epilepsy	Diagnosis AND prescription of anti-epileptics	G40–G41 [c]	N03	Twice in 1 year	0.267 (0.179–0.355)
29	Parkinson's disease	Diagnosis	G20–G22			0.166 (0.110–0.233)
30	Multiple sclerosis	Diagnosis	G35			0.366 (0.251–0.485)
31	Neuropathies	Diagnosis	G50–G64			0.049 (0.031–0.072)

ATC: Anatomical Therapeutic Chemical Classification System; ICD-10: International Statistical Classification of Diseases 10th Revision.

[a] Prescriptions are only used as indication in absence of previous ischemic heart disease and heart failure, and diuretics are furthermore only used as indication in absence of previous kidney disease.

[b] Prescriptions are only used as indication in absence of previous ischemic heart disease

[c] Amended in our application compared to the original study (1). The category *chronic pulmonary disease* in the original study have been divided into *chronic obstructive pulmonary disease* (ICD-10: J40–J44, J47) and *asthma* (ICD-10: J45–J46) as these diseases have very different disability weights. For cases ascertained only by medication use (ATC: R03), individuals with an age of onset below the median age of onset for asthma was considered as asthma cases, whereas individuals with an age of onset equal to or above this median was considered as chronic pulmonary disease cases.

Table S2. Persons at risk at start of follow-up and cases during follow-up in the total cohort of 162,483 individuals in four regions of Denmark, 2013-2022

Mental disorder category	Pre-existing cases before follow-up, N (%)	Persons at risk at start of follow-up, N (%)	Cases during follow-up/person-years at risk
**Total**			
Substance use disorders	1,364 (1.1)	161,119 (98.9)	330/1,027,108
Schizophrenia spectrum disorders	1,018 (0.9)	161,465 (99.1)	370/1,028,837
Bipolar disorder	658 (0.5)	161,825 (99.5)	231/1,031,241
Major depressive disorder	3,890 (2.8)	158,593 (97.2)	1,477/1,006,518
Anxiety or neurotic-related disorders	5,631 (4.2)	156,852 (95.8)	1,895/994,269
Personality disorders	1,581 (1.3)	160,902 (98.7)	392/1,024,885
Any of the included mental disorders	9,047 (6.6)	153,436 (93.4)	2,818/969,898
**Women**			
Substance use disorders	558 (0.8)	87,061 (99.2)	128/557,384
Schizophrenia spectrum disorders	537 (0.9)	87,082 (99.1)	199/557,139
Bipolar disorder	414 (0.6)	87,205 (99.4)	156/557,847
Major depressive disorder	2,650 (3.5)	84,969 (96.5)	936/540,839
Anxiety or neurotic-related disorders	3,750 (5.2)	83,869 (94.8)	1,212/533,096
Personality disorders	1,166 (1.8)	86,453 (98.2)	309/552,664
Any of the included mental disorders	5,811 (7.9)	81,808 (92.1)	1,736/518,283
**Men**			
Substance use disorders	806 (1.3)	74,058 (98.7)	202/469,724
Schizophrenia spectrum disorders	481 (1.0)	74,383 (99.0)	171/471,699
Bipolar disorder	244 (0.4)	74,620 (99.6)	75/473,395
Major depressive disorder	1,240 (2.0)	73,624 (98.0)	541/465,679
Anxiety or neurotic-related disorders	1,881 (3.2)	72,983 (96.8)	683/461,173
Personality disorders	415 (0.8)	74,449 (99.2)	83/472,222
Any of the included mental disorders	3,236 (5.4)	71,628 (94.6)	1,082/451,615

Absolute numbers are unweighted, whereas percentages are weighted based on register data to represent the population of the included regions in 2013 and 2017.

Table S3. Social disconnection and relative differences in incidence rates of seven mental disorder categories in four regions of Denmark, 2013-2022

		**Model 1**	**Model 2**
	Events/person-years at risk among exposed	*IRR (95% CI)*	*IRR (95% CI)*
**Loneliness**			
Substance use disorders	89/55,905	4.86 (3.58–6.59)	3.87 (2.80–5.34)
Schizophrenia spectrum disorders	118/55,990	5.04 (3.80–6.68)	4.14 (3.07–5.58)
Bipolar disorder	52/57,205	3.87 (2.66–5.62)	3.60 (2.48–5.21)
Major depressive disorder	280/51,297	3.32 (2.83–3.90)	2.94 (2.49–3.47)
Anxiety or neurotic-related disorders	411/48,351	3.82 (3.32–4.40)	3.22 (2.78–3.72)
Personality disorders	132/54,412	5.55 (4.27–7.21)	4.94 (3.78–6.47)
Any of the included disorders	536/43,628	3.62 (3.21–4.09)	3.15 (2.79–3.57)
**Social isolation**			
Substance use disorders	28/25,012	5.54 (3.36–9.13)	3.23 (1.96–5.34)
Schizophrenia spectrum disorders	30/25,187	7.70 (4.49–13.22)	4.80 (2.78–8.28)
Bipolar disorder	10/25,928	2.31 (1.05–5.06)	1.72 (0.78–3.83)
Major depressive disorder	78/24,178	3.13 (2.32–4.22)	2.48 (1.84–3.34)
Anxiety or neurotic-related disorders	101/23,511	4.73 (3.62–6.19)	3.21 (2.46–4.20)
Personality disorders	8/25,235	2.13 (0.94–4.83)	1.47 (0.64–3.39)
Any of the included disorders	125/21,368	3.62 (2.84–4.62)	2.68 (2.11–3.40)
**Low social support**			
Substance use disorders	106/131,487	2.91 (2.20–3.85)	2.45 (1.83–3.28)
Schizophrenia spectrum disorders	125/132,054	3.15 (2.40–4.14)	2.61 (1.95–3.48)
Bipolar disorder	46/133,173	1.43 (0.98–2.10)	1.32 (0.88–1.97)
Major depressive disorder	391/126,398	2.42 (2.11–2.78)	2.18 (1.89–2.51)
Anxiety or neurotic-related disorders	522/123,343	2.67 (2.36–3.02)	2.30 (2.02–2.62)
Personality disorders	124/130,529	3.07 (2.37–3.96)	2.82 (2.15–3.69)
Any of the included disorders	741/117,333	2.54 (2.29–2.81)	2.25 (2.02–2.50)
**Composite measure**			
Substance use disorders	151/169,995	3.78 (2.91–4.93)	3.16 (2.41–4.13)
Schizophrenia spectrum disorders	179/170,403	4.23 (3.27–5.47)	3.53 (2.70–4.61)
Bipolar disorder	78/172,329	2.39 (1.74–3.28)	2.25 (1.63–3.11)
Major depressive disorder	525/162,559	2.72 (2.39–3.09)	2.46 (2.15–2.81)
Anxiety or neurotic-related disorders	716/158,045	3.05 (2.72–3.41)	2.62 (2.33–2.96)
Personality disorders	185/168,531	4.21 (3.31–5.35)	3.85 (3.01–4.94)
Any of the included disorders	994/149,044	2.85 (2.59–3.13)	2.53 (2.30–2.78)

CI: Confidence interval; IRR: Incidence rate ratio. Missing data was imputed using multiple imputation by chained equations, and the results are weighted based on register data to represent the population of the included regions in 2013 and 2017. All estimates are adjusted for age, sex, year of survey participation, country of birth, educational level, income, and wealth (Model 2).

Table S4. Social disconnection and sex-stratified relative differences in incidence rates of seven mental disorder categories in four regions of Denmark, 2013-2022

	**Women**		**Men**	
	Events/person-years at risk among exposed	*IRR (95% CI)*	*Events/person-years at risk among exposed*	*IRR (95% CI)*
**Loneliness**				
Substance use disorders	34/33,859	3.92 (2.40–6.39)	54/22,046	3.87 (2.52–5.92)
Schizophrenia spectrum disorders	64/33,672	4.17 (2.85–6.11)	54/22,318	4.18 (2.67–6.55)
Bipolar disorder	41/34,144	3.83 (2.48–5.90)	11/23,060	3.12 (1.46–6.67)
Major depressive disorder	191/29,993	2.88 (2.34–3.54)	88/21,305	2.94 (2.20–3.93)
Anxiety or neurotic-related disorders	269/28,235	3.11 (2.60–3.72)	142/20,116	3.32 (2.62–4.21)
Personality disorders	100/32,036	4.36 (3.21–5.90)	32/22,376	7.35 (4.20–12.84)
Any of the included disorders	347/25,341	3.02 (2.59–3.53)	189/18,287	3.32 (2.72–4.07)
**Social isolation**				
Substance use disorders	10/11,737	4.19 (1.76–9.96)	18/13,276	2.86 (1.53–5.33)
Schizophrenia spectrum disorders	13/11,768	3.83 (1.70–8.66)	17/13,419	5.15 (2.55–10.40)
Bipolar disorder*	-	-	-	-
Major depressive disorder	42/10,948	2.38 (1.55–3.63)	36/13,231	2.54 (1.67–3.85)
Anxiety or neurotic-related disorders	48/10,697	2.84 (1.90–4.26)	53/12,813	3.37 (2.33–4.89)
Personality disorders*	-	-	-	-
Any of the included disorders	62/9,824	2.31 (1.62–3.30)	63/11,544	2.94 (2.11–4.10)
**Low social support**				
Substance use disorders	39/64,646	2.77 (1.70–4.52)	67/66,841	2.31 (1.61–3.32)
Schizophrenia spectrum disorders	67/64,689	2.78 (1.90–4.07)	58/67,365	2.47 (1.61–3.79)
Bipolar disorder	33/65,081	1.68 (1.04–2.72)	12/68,092	0.82 (0.39–1.70)
Major depressive disorder	245/60,680	2.26 (1.88–2.71)	146/65,718	2.07 (1.65–2.59)
Anxiety or neurotic-related disorders	316/59,041	2.32 (1.97–2.73)	207/64,303	2.27 (1.85–2.79)
Personality disorders	93/63,115	2.63 (1.93–3.58)	31/67,414	3.40 (2.00–5.80)
Any of the included disorders	438/55,577	2.31 (2.01–2.64)	303/61,756	2.18 (1.85–2.57)
**Composite measure**				
Substance use disorders	59/87,435	3.61 (2.32–5.63)	92/82,559	2.97 (2.11–4.18)
Schizophrenia spectrum disorders	93/87,266	3.59 (2.50–5.15)	86/83,137	3.49 (2.34–5.21)
Bipolar disorder	57/87,964	2.65 (1.79–3.90)	20/84,365	1.65 (0.89–3.05)
Major depressive disorder	338/81,379	2.56 (2.17–3.02)	186/81,180	2.28 (1.83–2.84)
Anxiety or neurotic-related disorders	444/78,757	2.59 (2.23–3.01)	271/79,288	2.66 (2.19–3.23)
Personality disorders	140/85,032	3.53 (2.66–4.68)	44/83,498	4.96 (2.97–8.26)
Any of the included disorders	603/73,636	2.53 (2.23–2.87)	390/75,409	2.52 (2.16–2.94)

CI: Confidence interval; IRR: Incidence rate ratio. Missing data was imputed using multiple imputation by chained equations, and the results are weighted based on register data to represent the population of the included regions in 2013 and 2017. All estimates are adjusted for age, year of survey participation, country of birth, educational level, income, and wealth (Model 2).
*Sex-stratified results for bipolar and personality disorders according to social isolation are not provided due to few cases.

Table S5. Social disconnection and age-stratified relative differences in incidence rates of seven mental disorder categories in four regions of Denmark, 2013-2022

	**16-45 years**		**>45 years**	
	Events/person-years at risk among exposed	*IRR (95% CI)*	*Events/person-years at risk among exposed*	*IRR (95% CI)*
**Loneliness**				
Substance use disorders	56/27,810	3.34 (2.23–5.01)	33/28,095	5.36 (3.14–9.16)
Schizophrenia spectrum disorders	97/27,468	4.01 (2.88–5.57)	21/28,522	4.65 (2.48–8.74)
Bipolar disorder	34/28,307	3.31 (2.09–5.24)	18/28,898	4.04 (2.23–7.33)
Major depressive disorder	192/25,064	2.84 (2.32–3.47)	87/26,233	3.18 (2.39–4.23)
Anxiety or neurotic-related disorders	288/22,942	3.10 (2.60–3.70)	123/25,409	3.70 (2.88–4.75)
Personality disorders	114/26,447	4.72 (3.55–6.29)	18/27,965	6.72 (3.41–13.27)
Any of the included disorders	382/20,501	3.00 (2.59–3.49)	154/23,127	3.63 (2.94–4.49)
**Social isolation**				
Substance use disorders	9/2,771	3.75 (1.69–8.31)	20/22,241	2.75 (1.44–5.25)
Schizophrenia spectrum disorders	16/2,741	5.50 (2.83–10.72)	14/22,446	3.07 (1.41–6.67)
Bipolar disorder*	-	-	-	-
Major depressive disorder	24/2,574	3.41 (2.07–5.63)	54/21,604	2.04 (1.44–2.90)
Anxiety or neurotic-related disorders	41/2,189	4.04 (2.73–6.00)	59/21,321	2.56 (1.79–3.68)
Personality disorders*	-	-	-	-
Any of the included disorders	48/1,763	3.93 (2.72–5.67)	78/19,604	1.97 (1.46–2.65)
**Low social support**				
Substance use disorders	63/46,546	2.39 (1.64–3.48)	44/84,942	2.63 (1.66–4.16)
Schizophrenia spectrum disorders	98/46,437	2.65 (1.92–3.66)	27/85,617	2.23 (1.24–4.02)
Bipolar disorder	31/47,115	1.62 (1.00–2.63)	15/86,059	0.80 (0.40–1.59)
Major depressive disorder	237/44,022	2.21 (1.84–2.66)	153/82,376	2.12 (1.71–2.64)
Anxiety or neurotic-related disorders	346/41,905	2.43 (2.07–2.84)	176/81,438	2.01 (1.60–2.51)
Personality disorders	104/45,486	2.80 (2.09–3.75)	20/85,043	2.97 (1.56–5.68)
Any of the included disorders	477/39,376	2.31 (2.02–2.64)	263/77,957	2.10 (1.77–2.50)
**Composite measure**				
Substance use disorders	87/62,076	2.82 (2.00–3.98)	63/107,918	4.06 (2.66–6.18)
Schizophrenia spectrum disorders	144/61,729	3.78 (2.80–5.11)	35/108,674	2.28 (1.31–3.99)
Bipolar disorder	50/62,909	2.40 (1.60–3.61)	28/109,420	1.86 (1.12–3.09)
Major depressive disorder	332/58,054	2.62 (2.21–3.11)	193/104,505	2.16 (1.76–2.65)
Anxiety or neurotic-related disorders	481/54,843	2.77 (2.39–3.21)	234/103,202	2.30 (1.88–2.82)
Personality disorders	158/60,349	3.83 (2.93–4.99)	27/108,182	3.76 (2.00–7.06)
Any of the included disorders	662/50,895	2.66 (2.36–3.00)	332/98,150	2.23 (1.89–2.62)

CI: Confidence interval; IRR: Incidence rate ratio. Missing data was imputed using multiple imputation by chained equations, and the results are weighted based on register data to represent the population of the included regions in 2013 and 2017. All estimates are adjusted for age, sex, year of survey participation, country of birth, educational level, income, and wealth (Model 2).
*Age-stratified results for bipolar and personality disorders according to social isolation are not provided due to few cases.

References

1. ISCED 2011 operational manual: guidelines for classifying national education programmes and related qualifications [Internet]. Paris: OECD Publishing; 2015. Available from: https://www.oecd-ilibrary.org/education/isced-2011-operational-manual_9789264228368-en

2. OECD. Framework for integrated analysis. In: OECD Framework for Statistics on the Distribution of Household Income, Consumption and Wealth [Internet]. Paris: OECD Publishing; p. 171–92. Available from: https://www.oecd-ilibrary.org/economics/oecd-framework-for-statistics-on-the-distribution-of-household-income-consumption-and-wealth/framework-for-integrated-analysis_9789264194830-11-en

3. Statistics Denmark. TIMES variabel - FAMDISPONIBEL_13 [Internet]. [cited 2023 Jun 30]. Available from: https://www.dst.dk/da/Statistik/dokumentation/Times/familieindkomst/famdisponibel-13

4. Statistics Denmark. TIMES variabel - FAMFORMREST_NY05 [Internet]. [cited 2023 Jun 30]. Available from: https://www.dst.dk/da/Statistik/dokumentation/Times/familieindkomst/famformrest-ny05

5. Momen NC, Østergaard SD, Heide-Jorgensen U, Sørensen HT, McGrath JJ, Plana-Ripoll O. Associations between physical diseases and subsequent mental disorders: a longitudinal study in a population-based cohort. World Psychiatry. 2024;23(3):421–31.

6. Kessing LV, Ziersen SC, Caspi A, Moffitt TE, Andersen PK. Lifetime incidence of treated mental health disorders and psychotropic drug prescriptions and associated socioeconomic functioning. JAMA Psychiatry [Internet]. 2023 Jul 12; Available from: https://jamanetwork.com/journals/jamapsychiatry/fullarticle/2806888

7. Bartlett JW, Harel O, Carpenter JR. Asymptotically unbiased estimation of exposure odds ratios in complete records logistic regression. Am J Epidemiol. 2015 Oct 15;182(8):730–6.

8. Hughes RA, Heron J, Sterne JAC, Tilling K. Accounting for missing data in statistical analyses: multiple imputation is not always the answer. Int J Epidemiol. 2019 Aug 1;48(4):1294–304.

9. Morris TP, White IR, Royston P. Tuning multiple imputation by predictive mean matching and local residual draws. BMC Med Res Methodol. 2014 Jun 5;14:75.

10. Wulff JN, Ejlskov L. Multiple imputation by chained equations in praxis: guidelines and review. Electron J Bus. 2017;15(1).

11. White IR, Royston P, Wood AM. Multiple imputation using chained equations: issues and guidance for practice. Stat Med. 2011 Feb 20;30(4):377–99.
